# Nanotechnology for the treatment of deep endometriosis: uptake of lipid core nanoparticles by LDL receptors in endometriotic foci

**DOI:** 10.6061/clinics/2019/e989

**Published:** 2019-07-03

**Authors:** Alessandra Bedin, Raul C Maranhão, Elaine R Tavares, Priscila O Carvalho, Edmund C Baracat, Sérgio Podgaec

**Affiliations:** I Departamento de Ginecologia e Obstetricia, Faculdade de Medicina (FMUSP), Universidade de Sao Paulo, Sao Paulo, SP, BR; II Laboratorio de Metabolismo de Lipides, Instituto do Coracao (InCor), Hospital das Clinicas HCFMUSP, Faculdade de Medicina, Universidade de Sao Paulo, Sao Paulo, SP, BR; III Hospital Israelita Albert Einstein, Sao Paulo, SP, BR

**Keywords:** Endometriosis, LDL Receptors, Nanotechnology, Cholesterol, Nanoparticles, Lipoproteins

## Abstract

**OBJECTIVE:**

Rapidly dividing cells in multiple types of cancer and inflammatory diseases undergo high low density lipoprotein (LDL) uptake for membrane synthesis, and coupling an LDL-like nanoemulsion, containing lipid nanoparticles (LDE) to a chemotherapeutic agent efficiently targets these cells without significant systemic effects. This was a prospective exploratory study that evaluated the uptake of a radioactively labeled LDE emulsion by receptors of endometriotic foci and the capacity of the LDE for cellular internalization.

**METHODS:**

The lipid profile of each patient was determined before surgery, and labeled LDE were injected into fourteen patients with intestinal or nonintestinal endometriosis. The radioactivity of each tissue sample (intestinal endometriosis, nonintestinal endometriosis, healthy peritoneum, or topical endometrium) was measured.

**RESULTS:**

The group with intestinal endometriosis presented higher levels of plasma LDL but lower LDE uptake by foci than the nonintestinal group, suggesting less cell division and more fibrosis. The uptake of LDE was highest in the topical endometrium, followed by the healthy peritoneum, and lowest in the endometriotic lesion. Since the endometriotic foci showed significant LDE uptake, there was likely increased consumption of LDL by these cells, similar to cells in cancers and inflammatory diseases. Plasma cholesterol levels had no influence on LDE uptake, which showed that the direct delivery of the nanoemulsion to target tissues was independent of serum lipoproteins. There were no significant differences in the parameters (*p*>0.01) because of the small sample size, but the findings were similar to those of previous studies.

**CONCLUSION:**

Nanotechnology is a promising therapeutic option for surgery and hormonal blockage for deep endometriosis, with a lower complication rate and no systemic side effects.

## INTRODUCTION

Endometriosis is a frequent gynecological disease that affects approximately 10% of women of reproductive age. This disease represents a considerable therapeutic challenge, especially with respect to deep endometriosis, in which lesion infiltration can exceed 5 mm; deep endometriosis occurs in up to 12% of patients 1-6.

The clinical treatment of deep endometriosis, an estrogen-dependent disease, is based on hormonal suppression. However, the results of this treatment are unsatisfactory since burdensome side effects that include climacteric symptoms, decreased bone mineral density, and irregular menstrual cycles are frequent; treatment is not effective in reducing endometriotic nodules, and symptoms often recur after treatment withdrawal 7-9.

The surgical approach is thus considered the gold standard for the treatment of deep endometriosis, but surgical complications, such as fistulas, hemorrhage, infection, intestinal subocclusion, bladder dysfunction or intestinal dysfunction, may be hazardous and even life threatening. Moreover, the effectiveness of surgery is also limited, with lesion recurrence rates of up to 20% and frequent postsurgical persistence of endometriotic foci 6,10-15. In this setting, new strategies for the treatment of deep endometriosis are mandatory, but the several therapeutic alternatives proposed to date have been proven to be ineffective or have not yet been tested in women 9,16-18.

Endometriosis is basically a proliferative and inflammatory disease. In rapidly proliferating tissues, accelerated mitosis requires high amounts of cholesterol to build new cell membranes. Thus, overexpression of low density lipoprotein (LDL) receptors occurs to take up LDL cholesterol into cells to meet rising cholesterol demands 19. The strong avidity of these cells for cholesterol allows the use of LDL for the purpose of effectively targeting chemotherapeutic drugs to proliferating tissues such as cancer and inflammatory tissues.

In pioneering studies, Maranhao et al. 20 showed that artificially made nanoparticles, termed lipid nanoparticles (LDE) and which resemble the structure of the native LDL, also have the ability to carry chemotherapeutic agents to target tissues, such as neoplastic, atherosclerotic or inflammatory tissues 20-29.

Recently, we showed that in fragments of deep endometriotic tissues excised from patients during surgery, LDL receptors were overexpressed. These results suggested that LDL uptake and, conceivably, LDE uptake by these tissues could be increased. Thus, this study aimed to investigate whether LDE are taken up by endometriotic tissues in women, which could lay the foundation for the use of an LDE drug carrier system as a new strategy to treat deep endometriosis.

## METHODS

The present pilot study was performed at the Gynecological Clinic at Hospital das Clínicas of the University of Sao Paulo Medical School (HC-FMUSP), where 14 patients were divided into two groups: one group comprised patients with intestinal endometriosis, and the other group comprised patients with deep endometriosis at other pelvic sites (nonintestinal endometriosis group).

The inclusion criteria were as follows: aged between 18 and 45 years; histologically proven ovarian or deep endometriosis; eumenorrheic menstrual cycles; and no use of hormone therapy, including gonadotropin-releasing hormone (GnRH) analogs, progestogens, and hormonal contraceptives, for three months prior to surgery.

All patients were clinically evaluated, and the indication criteria for surgical treatment of endometriosis were suspicion of obstructive ureteral or intestinal involvement (terminal ileus, appendix, or rectosigmoid with signs of subocclusion), ovarian endometriomas above 6.0 cm, or patients without clinical improvement (of pelvic pain) after 12 months of clinical hormonal treatment.

Prior to surgical intervention, the lipid profile of each patient in both groups was determined from serum samples obtained after a 12-hour fast. On the day prior to surgery, 0.1 ml of solid cholesterol lipid core nanoparticles (i.e., LDE) with cholesterol [^14^C]-oleate was injected intravenously into each patient in both groups.

On the day of surgery, a topical endometrial sample was collected using a Pipelle curette. Patients were subjected to videolaparoscopy for diagnostic confirmation of endometriosis, resection of lesions was performed, and a sample of healthy peritoneal tissue adjacent to one of the lesions of endometriosis was also obtained. The sites of the lesions were divided into ovary, retrocervical region, vagina, bladder, and rectosigmoid. Then, subjects were classified into intestinal and nonintestinal groups.

After the proposed surgical procedure, the pathologist and the surgeon, who followed all the procedures, selected samples of endometriotic tissues and healthy peritoneal tissues via macroscopic analysis of the surgical specimen. This same pathologist performed the endometrial timing to confirm the phase of the cycle of the patient. The topical endometrial tissue, the endometriotic foci, and the adjacent healthy peritoneal fragment were divided into two parts and separately sent for histopathological analysis and radioactivity analysis.

Triglycerides and cholesterol fractions were measured with the colorimetric-enzymatic method using commercial kits (Labtest, Sao Paulo, Brazil) or calculated using the Friedewald et al. (1972) formulas: LDL=total cholesterol - (VLDL+HDL) and VLDL=TGL/5.

The LDE were prepared according to the technique modified by Maranhao et al. 30. Lipids were extracted from tissue samples collected according to the conventional method described by Folch et al. 31 and then processed according to the method reported by Maranhao et al. 32, and radioactivity was measured with a liquid scintillation spectrometer (Packard 1600 TR, Palo Alto, CA).

The injected radiological dose was evaluated according to the standards of the International Commission on Radiological Protection (ICRP) 33.

The Kruskal–Wallis test 34 was used to compare the patients' characteristics and the results of the tests. The amount of the injected emulsion found at sites of interest standardized for 1 g was determined according to the group and compared between the groups using the Kruskal–Wallis test. Friedman's test or paired Wilcoxon's test was used to identify differences between sites for each group 34. The level of significance was 1% to correct for multiple comparisons. Spearman's correlations 34 were used to compare laboratory tests to the amount of fluid found at the sites of interest for the women with endometriosis to validate whether migration of the substance to these sites was influenced by laboratory test levels. Only nonparametric tests were used because of the small number of samples in each group, and therefore, there was no assumption about the data evaluated. The tests were performed with a significance level of 5%.

### Ethical Approval

The institutional review board approved the project (number: 435.641-10/16/13). The patients were previously informed about the research content, and all of them signed informed consent forms prior to the start of the study.

## RESULTS

The results of 14 patients were evaluated: 6 presented with intestinal endometriosis (intestinal endometriosis patients, IEP), and 8 presented with nonintestinal lesions (nonintestinal endometriosis patients, NIEP). Within this group, 1 patient had a retrocervical lesion, 1 had an ovarian endometrioma, 4 had retrocervical lesions and ovarian endometriomas, and 2 had retrocervical and bladder lesions. The mean age was slightly higher in the nonintestinal endometriosis group (39.6 years) than in the intestinal endometriosis group (36 years), with no statistically significant difference (*p*=0.136), as shown in Table 1. Similarly, no significant difference was observed in body mass index (BMI) between the different groups (*p*=0.730). With regard to the phase of the menstrual cycle, 3 patients from the intestinal endometriosis group and 5 from the nonintestinal group were in the follicular phase. For two patients, determining the phase of the cycle was not possible. 

The clinical presentation of endometriosis varied between groups. The patients with intestinal lesions (the IEP group) had dysmenorrhea, while 62.5% of the cohort had no bowel lesions (NIEP group). Deep dyspareunia was reported in 100% and 87.5% of IEPs and NIEPs, respectively, but no differences were observed between the endometriosis groups concerning chronic pelvic pain or infertility (66.7% *versus* 62.5% and 50.0% *versus* 50.0%, respectively, for the IEP and NIEP). Cyclic intestinal alteration was reported in 66.7% of IEP and 25.0% in NIEP. Cyclic urinary alterations were more common in NIEP than in IEP (50% and 16.7%, respectively).

Table 2 shows the laboratory parameters evaluated in this study. The nonintestinal endometriosis group showed a tendency toward having lower total cholesterol, LDL cholesterol, and triglycerides. No significant differences were observed between the groups (*p*>0.05).

Table 3 shows the comparison of the LDE uptake level in the different tissues of both groups (intestinal endometriosis and nonintestinal endometriosis). In the statistical analysis of these values, no significant difference in the amount of LDE uptake was found between the groups (*p*>0.01).

**Figure 1:**
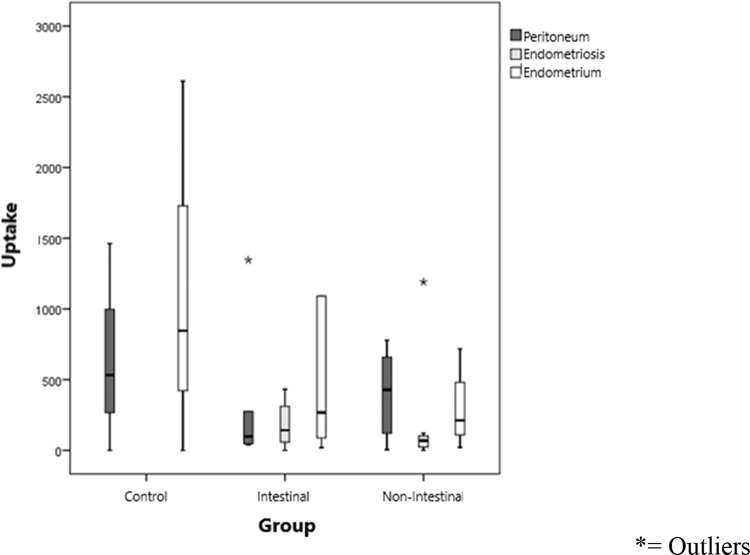
Uptake values of the labeled lipid nanoparticles (LDE) of each group (control, intestinal endometriosis, and nonintestinal endometriosis) and of the site evaluated (peritoneum, endometrium, and endometriosis) in each group.

Figure 1 shows the uptake values of the labeled lipid core nanoparticles (LDE), divided by group (IEP and NIEP) and uptake according to the site evaluated (peritoneum, endometrium, and endometriosis). Endometrial tissues showed uptake of the LDE in a similar manner. Although not statistically significant, uptake was highest in the endometrium, followed by the peritoneum and, finally, the endometriotic tissue (*p*>0.01).

Spearman's correlations 34 were calculated between laboratory tests and the amount of fluid found at sites of interest to verify whether migration of the substance to these sites was influenced by the levels in laboratory test results. In comparisons of the level of nanoparticle uptake at the different sites evaluated with the results of the laboratory tests of the patients (Table 4; r value), no statistically significant difference was found between the parameters (Table 4; *p*>0.05).

## DISCUSSION

Surgical treatment of deep endometriosis remains the most effective therapeutic option. However, technical difficulties are often encountered, and complications can be serious. The rates of different complication types vary between studies, but the most common complications are recurrent lesions (5.8% to 20%), fistulas (1.8% to 2.6%), hemorrhage (2.0 to 2.5%), infection (0.6% to 0.8%), intestinal subocclusion (0.2 to 2.7%), and bladder and/or intestinal dysfunction (3.6 to 9.8%) 6,10-15. In addition, surgery depends on specialized teams for cases of greater complexity, demonstrating the need for other alternatives to control deep endometriotic nodules, as well as clinical symptomatology.

LDL is the largest cholesterol transporter of the body 35,36, and tumor cells have increased LDL uptake compared to normal cells 32,35,37-40. This phenomenon occurs due to the increased need for cholesterol by tumor cells for membrane synthesis in cell growth and division 41. This is the main motivation for investigating LDL as a specific transport vehicle for antineoplastic drugs; the transport vehicle is developed by incorporating alternative molecules in place of the original molecule and substituting its internal lipids with other hydrophobic compounds 42 to limit the side effects of the drug and prevent the development of drug resistance 43.

Currently, chemotherapeutic agents, such as paclitaxel, carmustine, and etoposide, are being made to be nearly devoid of systemic toxicity when carried in an LDE, an artificial lipid emulsion similar to the LDL that has been demonstrated in experimental animals and patients with advanced cancers and atherosclerosis and has shown a good capacity for cellular destruction 20-22,26-29. LDE make it possible to use LDL receptor-mediated endocytosis in clinical practice because, unlike the native LDL, LDE preparations of drugs stably associated with LDE can be amenable to being manufactured at an industrial production scale.

Endometriosis may have a pathophysiological correlation with cancer due to similar mechanisms of growth and invasion 44 and to higher plasma LDL levels in endometriosis patients than in normal controls 45,46, which is also observed for some types of cancer 39,47. The overexpression of LDL receptors on the cell membranes of endometriotic lesions, compared to the topical endometrium, suggests the increased need for LDLs for cell membrane formation in dividing cells 19, a principle that also applies to cancer.

The present study was a pilot study that aimed to validate whether these LDL receptors, overexpressed in endometriotic foci, could take up the labeled LDE. The small sample size, namely, 14 patients, was comparable to those of previous similar studies (Maranhao et al. 20: 42 patients; Azevedo et al. 23: 4 patients; Pinheiro et al. 24: 16 patients; Pires et al. 26: 5 patients; and Graziani et al. 27: 14 patients). The control group comprised the other evaluated tissues (peritoneum and normal endometrium). The control group of subjects is necessary in the second stage of a study, when there would be a drug coupled to the nanoparticle to treat the disease.

The “Annual Limit for Intake” (ALI) parameter of a radionuclide is defined as the amount of radioisotope that induces an equivalent dose of 50 mSv. For organic components labeled with ^14^C, the ALI values are 9×10^7^ Bq 33. In the present study, the injected dose of ^14^C was 22.2×10^4^ Bq, which equals (22.2×10^4^ Bq/9×10^7^ Bq)×50 mSv=0.12233 msV. The equivalent dose incorporated into the whole body as a consequence of exposure to radioactive lipids is estimated to be 0.04 mSv, as assessed by the Medical Internal Radiological Dosimetry (MIRD) method 48. This value is within the mean described in laboratory evaluations of these parameters in humans 49. The method described above allowed us to estimate the injected dose of ^14^C cholesterol oleate in the participants of this study: 0.26 mGy in the lower large intestine, 0.5 mGy in the upper large intestine, 0.18 mGy in the skin, 13 mGy on the bone surface, and 0.13 mGy in the liver. The dose administered to the lungs, heart, and ovaries was insignificant. The dose-equivalent incorporated into the whole body, as a consequence of exposure to radioactive lipids, was estimated to be 0.02 mSv as assessed using the MIRD method 32. The maximum dose of radioactivity exposure for the general public (nonoccupational dose), according to the radiation protection guidelines, is 5 mSv per year 50. Comparatively, a chest X-ray is equivalent to 0.1 mSv, which is the dose-equivalent incorporated into the whole body; a full-body computed tomography scan is equivalent to 11 mSv; and an air travel time of 10 hours is equivalent to 0.05 mSv. Thus, previous studies were ethically approved, since nanoparticle biosafety has been demonstrated in both target and healthy control groups 32,33,51-53; therefore, the risk of harmful radioactive effects is not significant.

The comparisons between groups (intestinal and nonintestinal endometriosis; Table 1) showed no significant differences in the clinical parameters (*p*>0.01), including age and menstrual cycle phase, indicating that the groups were clinically for comparisons. Despite this finding, the group with intestinal endometriosis presented a lower BMI, comparable to the meta-analysis of Zhu et al. 54, where despite the heterogeneity of the groups analyzed, the presence and severity of endometriosis were inversely proportional to BMI. This difference, although not statistically significant since this was a pilot study with a small sample size, could also be observed in relation to symptomatology (Table 2): women in the control group had no symptoms of endometriosis, and those in the intestinal endometriosis group were more symptomatic in terms of pain than those with nonintestinal endometriosis. Respectively, in the intestinal and nonintestinal endometriosis groups, 87.5% and 62.5% of patients had dysmenorrhea, 66.7% and 62.5% of patients had chronic pelvic pain, and 66.7% and 25.0% of patients complained of a cyclic intestinal disorder. In contrast, women with nonintestinal endometriosis had more symptoms of deep dyspareunia (100% *versus* 87.5% of those with intestinal endometriosis) and cyclical urinary changes (50% *vs*. 16.7%). These findings support the data from other studies, where deep endometriosis, with extensive involvement, was observed to correspond to cases of greater symptomatology 1,55-57.

Regarding the level of cholesterol, which can indirectly demonstrate a high metabolism status due to inflammation and a high level of cell division, the group with intestinal endometriosis presented the highest levels of LDL: 121 mg/dL (control, 112 mg/dL, and nonintestinal endometriosis, 87 mg/dL, *p*>0.01; Table 3). These results confirm those of previous studies, which suggest hypercholesterolemia in women with endometriosis 45,46, and larger groups are recommended for this evaluation.

In Table 3, LDE uptake in different tissues of each group was compared. The peritoneum tended to take up more LDE in women with nonintestinal endometriosis (mean of 712.3 U; *p*>0.1). In lesions of nonintestinal endometriosis, the mean uptake level was greater than that in lesions of intestinal endometriosis (*p*>0.1), showing greater LDL internalization, which suggests fewer fibrotic tissues in these cases. In the topical endometrium, LDE emulsion uptake in the intestinal endometriosis group (1340.4 cpm/g) was higher than that in the nonintestinal endometriosis group (709.9 cpm/g, *p*>0.1). Despite this result, in the statistical analysis of these values, no significant difference was observed in the level of nanoparticle uptake between the different tissues (*p*>0.01). Moreover, the comparison of LDE uptake by each site in the evaluation (healthy peritoneum, endometriotic lesion and topical endometrium) did not show any statistically significant difference between the groups (Figure 1), despite the observation of decreasing uptake by the topical endometrium (1067.5 cpm/g), healthy peritoneum (564.7 cpm/g), and endometriotic lesion (189.2 cpm/g). Since this is a pioneering study, no data from the literature are available with regard to the uptake of the LDE in endometriosis for comparison.

However, the result that endometriotic foci (intestinal or not) significantly take up LDE suggests increased LDL consumption in endometriotic lesions, as well as in cancer and inflammatory diseases, where LDE was tested as drug carriers 20-26.

The results of this study are preliminary but show high uptake of labeled LDE by the topical endometrium (Tables 4 and 5). One possible interpretation of this result is that the topical endometrium is a tissue with an increased rate of cell division, since it proliferates, thickens, and desquamates cyclically in a short period of time. Such cyclic renewal of all endometrial tissue also suggests that the eventual uptake of the drug by the same mechanism would not compromise endometrial function, since cellular apoptosis would happen independently of the treatment, and new cells would appear soon after. Nevertheless, future studies are necessary for this evaluation, and women with no reproductive aspirations should be selected first. Further studies should also be carried out to evaluate other possibilities of treatment side effects, such as alterations to the ovarian reserve, with a blocking drug coupled to an LDE.

In the present study, the possibility of labeled LDE uptake by the tested tissues having an influence on plasma cholesterol levels (Table 4) was also evaluated, and no significant differences among the different groups/tissues was found. This confirms the direct delivery of the nanoemulsion to the tissues with high levels of cell division, independent of serum lipoproteins.

In conclusion, ^14^C-labeled LDE were taken up by the endometriotic tissues of patients with ovarian and deep endometriosis, by the healthy peritoneum adjacent to the lesions, and by the endometrium. In the nonintestinal endometriotic lesions, the mean uptake value was greater than that in the intestinal endometriosis lesions.

These results can lead to the development of new studies evaluating nanotechnology as a therapeutic option for radical surgeries and their complications. In addition, due to the lack of systemic side effects, when this technology is compared to systemic hormonal blocks that are currently available as a therapeutic option, further studies will be motivated to attempt to develop a more effective treatment with fewer complications than the treatments that are currently available or to develop an adjuvant treatment to lower surgical invasiveness.

## AUTHOR CONTRIBUTIONS

Bedin A assisted in the design of the study, conceptualized the manuscript and wrote the first draft, took the lead on subsequent edits, performed the surgical procedure and collected biological material. Maranhão RC assisted in the design of the study, assisted in conceptualizing the manuscript, oversaw the statistical analysis and provided substantive edits on the analytic plan and manuscript. Tavares ER performed biochemical and statistical analysis and provided substantive edits to the manuscript. Carvalho PO performed biochemical and statistical analysis and provided substantive edits to the manuscript. Baracat EC assisted in the design of the study, assisted in conceptualizing the manuscript, oversaw the statistical analysis and provided substantive edits on the analytic plan and manuscript. Podgaec S assisted in the design of the study, assisted in conceptualizing the manuscript, oversaw the statistical analysis and provided substantive edits on the analytic plan and manuscript.

## Figures and Tables

**Table 1:** Clinical parameters are expressed as the mean and standard deviation of the different groups: without endometriosis, with intestinal endometriosis, and with endometriosis in other sites (nonintestinal).

Variable	Group			
	Control (CG) (n=3)	Intestinal (IEP) (n=6)	Nonintestinal (NIEP) (n=8)	*p*
Age (years)	30.3±2.9	36±9.4	39.6±5.8	0.136
Weight (kg)	75.1±25	67.8±12.2	74.5±14.7	0.788
Body mass index (kg/m2)	28.8±7.0	27.3±4.2	28.8±3.7	0.730

Kruskal-Wallis test.

**Table 2:** Results for total cholesterol concentrations, their fractions and triglyceride levels in mg/dL (average and standard deviation) of the three groups of patients: no endometriosis, intestinal endometriosis, and endometriosis in other sites (nonintestinal).

Variable (mg/dL)	Group			
	Control group (CG) (n=3)	Intestinal (IEP) (n=6)	Nonintestinal (NIEP) (n=8)	*p*
Total cholesterol	226±49	198±35	154±53	0.26
HDL cholesterol	45±14	47±3	46±15	0.83
LDL cholesterol	112±26	121±24	87±40	0.21
Triglycerides	115±20	155±59	107±40	0.29

Kruskal-Wallis test; mg/dL, milligrams per deciliter.

**Table 3:** Uptake of labeled nanolipid emulsion at the sites of interest (peritoneum, endometriosis and endometrium), expressed as the mean and standard deviation, according to patient group, namely, no endometriosis, intestinal endometriosis, and endometriosis in other sites (nonintestinal), and comparative test results.

Variable (cpm/g)	Group			
	Control (n=3)	Intestinal (n=6)	Nonintestinal (n=8)	p*
Peritoneum	665 (739.9)	316.7 (511.9)	712.3 (1069.1)	0.538
Endometriosis	-	180.8 (169.8)	197.5 (403.5)	0.651
Endometrium	1152.3 (1332.2)	1340.4 (2220.4)	709.9 (1275.5)	0.941

* Kruskal-Wallis test. Due to the multiple comparisons, the tests were analyzed with a significance level of 1% (α=0.01). Cpm/g (radioactive count/tissue gram).

**Table 4:** Values of correlations between the levels of nanoparticles found at the sites of interest (peritoneum, endometriosis, and endometrium), concentrations of total cholesterol and its fractions, and levels of triglyceride in patients with endometriosis.

Correlation		Peritoneum	Endometriosis	Endometrium
TC	r	0.321	0.333	0.286
	*p*	0.365	0.347	0.493
	n	10	10	8
HDL	r	0.201	0.237	0.143
	*p*	0.578	0.510	0.736
	n	10	10	8
LDL	r	0.261	0.176	0.143
	*p*	0.467	0.627	0.736
	n	10	10	8
TGL	r	-0.261	-0.188	-0.405
	*p*	0.467	0.603	0.320
	n	10	10	8
Spearman’s correlation		

r=uptake rate with serum parameter; *p*=*p* index; n=number of patients.
